# Asphyxia Neonatorum

**Published:** 1910-07-23

**Authors:** 


### Gynæcology.

### ASPHYXIA NEONATORUM.

The birth of a child in a condition of suspended animation is a common occurrence and often requires prompt treatment. Many infant lives are lost annually through an insufficient recognition of this, especially in those cases in which the child is pale when born instead of cyanosed. It is usual to classify these cases according to the appearance of the child: if it is blue and its heart is beating strongly, it is known as asphyxia livida; if it is pale and the heart-beat is only just recognisable, we call the condition asphyxia pallida. The former is much more common, and of course is due to imperfect blood aeration during the last few minutes of labour. It is caused by any condition which interferes with the complete interchange of gases in the placenta between the maternal and the fœtal blood. The most important cause is, therefore, premature separation of the placenta, such as occurs in antepartum hæmorrhage and in breech cases, where the head is delayed in the lower uterine segment or the vagina. In the latter condition the upper contracted portion of the uterus may separate the placenta before the head is delivered. Pressure on the cord is the time-honoured explanation of these cases, but is not in reality so frequent an occurrence. The cord can only be compressed between the fœtal head and the pelvic brim, and the relative shapes of these two structures is such that there must be plenty of room for the cord all round except opposite the promontory of the sacrum or the pubic symphysis. Stretching of the cord when wound tightly round some part of the fœtus may lead to such a narrowing of the lumen of its vessels as to interfere with the circulation. Tonic contraction of the uterus is an uncommon cause, but acts by emptying the placental vessels on account of the continuous pressure which it exerts. Premature efforts at respiration, such as we see sometimes in breech cases, leads to the inspiration of blood mucus and liquor amnii into the fœtal bronchi, and consequently asphyxia supervenes as soon as the placental circulation ceases, because inspired air is thereby prevented from reaching the air vesicles. This cause is more likely to become operative after birth, for if the placental circulation is intact it makes no difference whether the bronchi are blocked with fluid or not. In blue asphyxia the skin is characteristically coloured, the skin reflexes are preserved, and the heart-beats can be seen, as well as felt, through the chest-wall.

Asphyxia pallida, on the other hand, is a much more serious condition, and its causation is not always the same. In some cases it is really the final result of a condition of deficient blood aeration due to the same causes as those mentioned for blue asphyxia, the pallor of the skin being due to a failing circulation with a general lowering of the blood-pressure. This, however, is not always the case, for white asphyxia may occur in deliveries in which there is no interference with the placental circulation. For instance, it may occur in difficult forceps deliveries in vertex presentations, in which case the circulatory failure is primarily due to some injury or interference with the cardiac and respiratory centres. The pale skin is then due to emptying of the vessels by the general compression which the fœtus undergoes during delivery, the feebly acting heart being unable to fill them up again at once. In such cases the blood collects in the abdominal organs, the portal area in particular, and the condition is closely allied to shock such as occurs after serious operations and long anæsthetics in adults. In such circumstances the child is colourless, the muscles are all relaxed, the skin reflexes are entirely lost, and the heart beats so feebly that it may only be recognisable by auscultation. The heart must beat, however, for the condition to merit the term asphyxia pallida, for if the heart has ceased to beat the child is dead.

The treatment of these two forms of asphyxia is very different, on account of the feeble circulation and loss of reflexes in the pallid form. Indeed, it is a fact that most infants in a condition of blue asphyxia with a vigorous heart-beat will recover by themselves if left alone. The venous blood circulating through the brain is usually sufficient to excite the respiratory centre and cause efforts at respiration. It is not advisable, however, to leave such infants alone, for if respiration is not easily and quickly established, treatment may be applied too late to bring this about. It is not necessary to hurry the separation of the infant from the placenta, but the first effort should be made to clear the mouth and throat of any fluids which may have been sucked in. It is rarely necessary to attempt to remove fluid from the bronchi, but if the infant has been seen to make efforts at respiration before delivery, this should be done. It is simple to pass a soft catheter through the larynx into the trachea and then apply gentle suction, or to blow a little air into the trachea with a ball syringe. This may have the effect of displacing the fluid, which will then flow out into the mouth. This procedure may, however, do harm unless carried out with great gentleness, for the air vesicles may be ruptured in the process and emphysema may thus arise. As a rule all that is necessary is to wipe out the mouth and throat, holding the child up by the feet to assist the outflow of fluids. After this the skin of the infant may be stimulated by rubbing the back with a towel, or sprinkling a little cold water on it. If no response follows this treatment, the cord should be tied and cut and the child immersed in a warm bath and a cold bath alternately. As a rule respiration is started by these measures, but if it is not, artificial respiration must be resorted to without further delay.

In the pallid form it is useless to attempt to reflexly excite the respiratory centre because all skin reflexes are lost, consequently artificial respiration must be used at once. It is better, therefore, to tie and cut the cord immediately, and place the child in a warm bath. The reason for the bath is that the failing circulation allows of rapid cooling of the skin, and this can be prevented by applying artificial warmth. Artificial respiration can be carried out whilst the child is in the bath, if Sylvester's method or chest compression is the method made use of. In fact, these are the only means which can be used whilst the child is in a bath, unless we employ Laborde's tongue traction method, which has never been much used in this country. In future, probably, Schafer's new method will be employed for these infants with better hope of success, provided the child can be kept warm by surrounding it with flannels and placing it face downwards upon an indiarubber hot-water bottle. A warm rectal injection of an ounce of saline solution with five minims of brandy may occasionally help to stimulate the heart-beat. It is important to remember that artificial respiration should be used in such a way as not to interfere with any spontaneous efforts of the child to breathe; in fact, if the child does make spontaneous efforts, it is better to leave off artificial respiration. When respiration is established, the child should be kept very warm, and carefully watched so that any secondary respiratory failure may be at once dealt with.

